# Corneal Confocal Microscopy as a Quantitative Imaging Biomarker of Diabetic Peripheral Neuropathy: A Review

**DOI:** 10.3390/jcm11175130

**Published:** 2022-08-31

**Authors:** Eleonora Cosmo, Giulia Midena, Luisa Frizziero, Marisa Bruno, Michela Cecere, Edoardo Midena

**Affiliations:** 1 IRCCS—Fondazione Bietti, 00198 Rome, Italy; 2 Department of Neuroscience-Ophthalmology, University of Padova, 35128 Padova, Italy

**Keywords:** corneal confocal microscopy, diabetes, diabetic peripheral neuropathy

## Abstract

Distal symmetric polyneuropathy (DPN), particularly chronic sensorimotor DPN, represents one of the most frequent complications of diabetes, affecting 50% of diabetic patients and causing an enormous financial burden. Whilst diagnostic methods exist to detect and monitor this condition, they have significant limitations, mainly due to their high subjectivity, invasiveness, and non-repeatability. Corneal confocal microscopy (CCM) is an in vivo, non-invasive, and reproducible diagnostic technique for the study of all corneal layers including the sub-basal nerve plexus, which represents part of the peripheral nervous system. We reviewed the current literature on the use of CCM as an instrument in the assessment of diabetic patients, particularly focusing on its role in the study of sub-basal nerve plexus alterations as a marker of DPN. CCM has been demonstrated to be a valid in vivo tool to detect early sub-basal nerve plexus damage in adult and pediatric diabetic patients, correlating with the severity of DPN. Despite its great potential, CCM has still limited application in daily clinical practice, and more efforts still need to be made to allow the dissemination of this technique among doctors taking care of diabetic patients.

## 1. Introduction

Diabetes mellitus is one of the most common chronic diseases worldwide, with an estimated global prevalence among the adult population of 10.5% [1]. Its prevalence will continue to increase in the next few decades, and it is expected to rise to 12.2% in 2045, carrying an enormous financial burden mainly due to the complications of the disease [2].

Both Type 1 diabetes mellitus (T1DM), characterized by absolute insulin deficiency due to the loss of the secretory function of pancreatic β-cells, and Type 2 diabetes mellitus (T2DM), in which there is a progressive loss of insulin secretion on the background of insulin resistance [3], are accompanied by characteristic macrovascular and microvascular complications, the latter including retinopathy, nephropathy, and neuropathy [4]. 

Diabetic neuropathies represent a heterogeneous group of disorders that could be classified into generalized symmetric polyneuropathies (i.e., acute sensory, chronic sensorimotor, and autonomic) and focal and multifocal neuropathies (i.e., cranial, truncal, focal limb, proximal motor or amyotrophy and coexisting chronic inflammatory demyelinating polyneuropathy), according to the affected part of the nervous system and to the clinical presentation [5]. Among diabetic neuropathies, distal symmetric polyneuropathy (DPN) and, in particular, chronic sensorimotor DPN represents the most frequent disorder, since it affects approximately 50% of diabetic patients. It is defined as a progressive loss of peripheral nerve axons in a distal to a proximal pattern, leading to complications such as pain, reduced sensitivity, and foot ulceration [6]. 

Corneal confocal microscopy (CCM) is an in vivo technique that allows, in a non-invasive way, the study of all corneal layers, including the sub-basal nerve plexus, which represents part of the peripheral nervous system. In particular, corneal nerves arise from the ophthalmic branch of the trigeminal nerve, containing myelinated Aδ-fibers that lose their myelin sheath within 1 mm of the limbus to guarantee corneal transparency and unmyelinated C fibers. They enter the middle stroma and then track anteriorly reaching and penetrating the Bowman’s layer, where they spread in a network of fibers running parallel to the cornea surface forming the sub-basal plexus [7].

Our group previously demonstrated how CCM could represent an in vivo, non-invasive, and reproducible diagnostic technique [8] for the study of corneal morphological alterations not only in diabetic patients but also in other conditions such as post-refractive surgery [9,10], topical chemotherapy in ocular surface squamous neoplasia [11], vernal keratoconjunctivitis [12], Wilson disease [13], oxaliplatin-induced peripheral neuropathy [14], therapy with epidermal growth factor receptor (EGFR) inhibitor depatuxizumab mafodotin (ABT-414) in patients affected by EGFR-amplified recurrent glioblastoma [15,16], and recently in COVID-19 recovered patients [17].

In the last decade, multiple studies conducted on diabetic patients have provided evidence indicating that morphological changes in the sub-basal nerve plexus strongly correlate with peripheral nerve damage and, thus, with DPN. In consequence, CCM is currently considered a reliable, repeatable, and quantitative diagnostic method in the screening, diagnosis, and monitoring of DPN.

Our purpose was to review the current literature on the use of CCM as an instrument in the assessment of diabetic patients, focusing on its role in the study of sub-basal nerve plexus alterations as a marker of DPN, particularly chronic sensorimotor DPN. Hence, the findings reported in this review regarding diabetic peripheral neuropathy refer to chronic sensorimotor distal polyneuropathy.

To identify potentially relevant articles in the medical literature, we searched MEDLINE for English language articles published from January 1995 to May 2022. MEDLINE was queried using the following search terms (used both individually and in combination for advanced research): corneal confocal microscopy, diabetes, and diabetic neuropathy. Additional articles were identified by reviewing the references of examined publications. To identify potentially relevant articles to include in this review, two investigators reviewed each paper. Articles included in the reference list were fully examined by the authors.

## 2. Principles of Confocal Microscopy 

Marvin Minsky developed the original confocal microscope (CM) in 1955 [18], but it was only in 1985 that this technology was used to analyze ex vivo the corneal morphology [19], while the first in vivo images of the human cornea were published by Cavanagh et al. in 1990 [20]. 

The difference between a CM and a conventional light microscope is the poorer image quality of the latter due to reflections and light scattered from the structures that are outside the focal plane of the microscope [21]. CM overcomes the problem of defocused light thanks to the confocal principle: A single point of tissue can be illuminated by a point light source and simultaneously imaged by a camera in the same plane, providing images with a very high resolution and magnification and an en-face view of the structure under evaluation [22].

There are three types of corneal confocal microscopes that differ in factors such as the type and intensity of the light source, magnification, image contrast, and image resolution. They are, from the oldest to the more recent, tandem scanning confocal microscopes (e.g., Confoscan P4, Tomey Corporation, Cambridge, MA, USA), slit scanning confocal microscopes (e.g., Confoscan 4, Nidek Technologies, Japan), and laser scanning confocal microscopes (e.g., Heidelberg Retina Tomograph III Rostock Corneal Module, Heidelberg, Germany) [23]. Differently from the others, a laser scanning confocal microscope has the ability to serially produce images of thin layers from the cornea since it has a smaller depth of focus, thus providing more accurate imaging of the cornea [24]. For this reason, the Heidelberg Retina Tomograph III Rostock Corneal Module (Heidelberg, Germany) is the most used device in recent studies, approximately since 2010, evaluating the corneal sub-basal nerve plexus not only in the diabetic population but also in other systemic disease affecting the peripheral nerves.

## 3. The Sub-Basal Nerve Plexus Observed through Different Parameters

**Figure 1:**
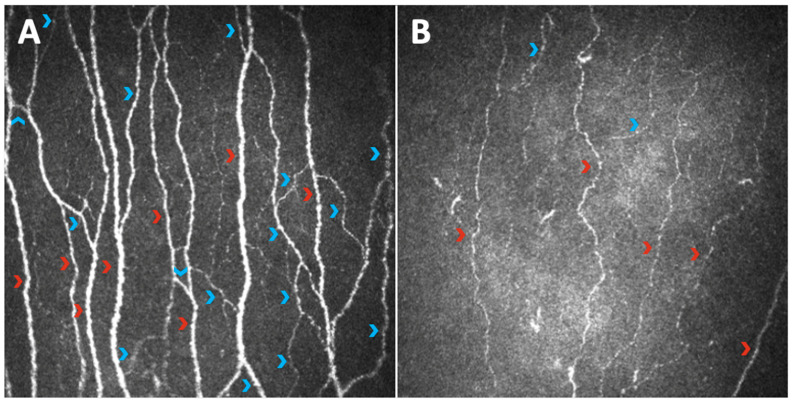
Images of the sub-basal nerve plexus captured with Heidelberg Retina Tomograph III Rostock Corneal Module (Heidelberg, Germany) from the central cornea of a healthy subject (**A**) and a diabetic subject with diabetic peripheral neuropathy (**B**). The comparison clearly shows the paucity of corneal nerve fibers in the diabetic neuropathic patient; in particular, there is evidence of a reduction in both the main fibers (red arrowheads) and the branches (blue arrowheads), determining a decrease in corneal parameters of length and density such as CNFL, CNFD, and CNBD.

In 2000, Rosenberg was the first to study corneal structure in T1DM patients by means of CCM, demonstrating that sub-basal nerve fiber density was reduced in patients versus healthy controls and that this decrease correlated with DPN severity [25]. A few years later, other works began to further discuss the same matter, suggesting that CCM could provide a means to study human diabetic neuropathy in clinical trials [26,27]. The following three parameters were first developed as potential indicators of corneal nerve fiber status: corneal nerve fiber density (CNFD—the total number of major nerves per mm^2^ of corneal tissue), corneal nerve fiber length (CNFL—the total length of all nerve fibers and branches; mm per mm^2^ of corneal tissue), and corneal nerve branch density (CNBD—the number of branches emanating from major nerve trunks per mm^2^ of corneal tissue); the latter, in particular, represents the regenerative capacity of this nervous plexus [26]. They were all reduced in a diabetic population versus healthy controls, with a tendency for greater reduction with increasing severity of neuropathy (Figure 1).

**Figure 2:**
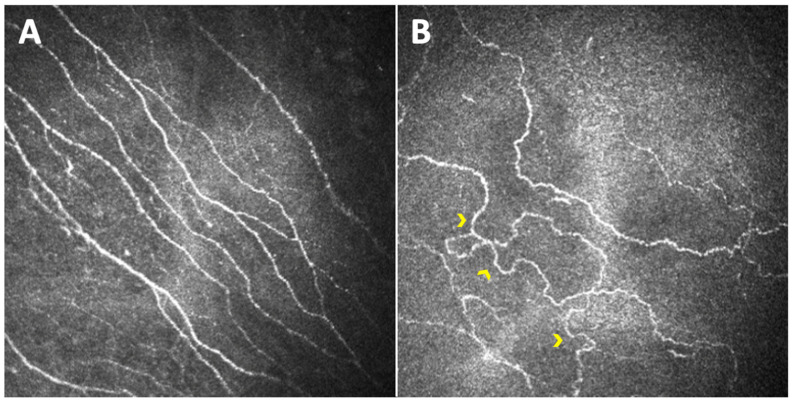
Images of the sub-basal nerve plexus captured with Heidelberg Retina Tomograph III Rostock Corneal Module (Heidelberg, Germany) from the central cornea of a healthy subject (**A**) and a diabetic subject with diabetic peripheral neuropathy (**B**). While the corneal nerve fibers of the healthy subject are mostly straight, those of the diabetic neuropathic patient show increased tortuosity (yellow arrowheads).

In the work of Kallinikos et al., another parameter was introduced in the assessment of diabetic corneal neuropathy: nerve fiber tortuosity [28]. It appeared to be significantly greater in a group of diabetic patients with severe neuropathy than in healthy control subjects, and in the mild and moderate neuropathic groups (Figure 2). The authors suggested that an increase in nerve tortuosity may represent a morphologic marker of nerve regeneration. 

**Figure 3:**
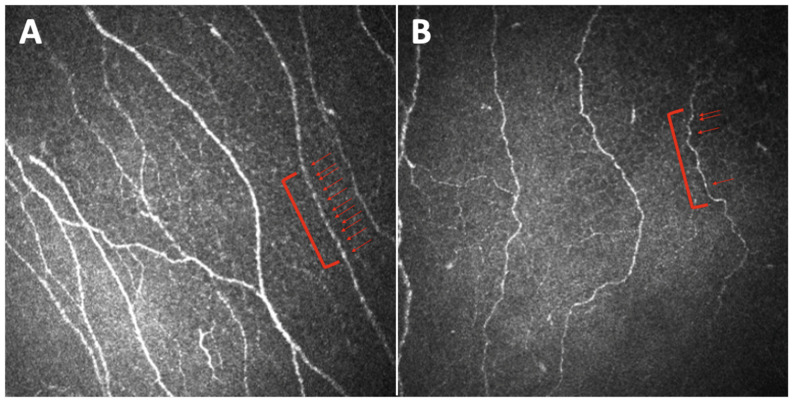
Images of the sub-basal nerve plexus captured with Heidelberg Retina Tomograph III Rostock Corneal Module (Heidelberg, Germany) from the central cornea of a healthy subject (**A**) and a diabetic subject with diabetic peripheral neuropathy (**B**). Red arrows indicate nerve beadings, counted in 100 µm of a single nerve fiber (red lines). The comparison highlights how nerve beadings are reduced in diabetic neuropathic patients versus healthy subjects.

Midena et al. first reported alterations in a new corneal parameter in diabetic patients, since they found, in patients versus healthy controls, a reduction in the number of nervous beadings with a decreasing trend among patients with an increasing grade of neuropathy (Figure 3) [29]. These authors concluded that, since nerve beadings document the metabolic activity of the corneal sub-basal nerve plexus, representing an accumulation of mitochondria along the nerve, their significant decrease mirrors the pathologic metabolic activity of diabetic small nerve fibers. Nerve beadings were also studied by Ishibashi et al., who reported in a T2DM population, beyond a reduction in the frequency of beadings, an increase in the bead size, in particular in patients with severe neuropathy [30]. 

Other parameters that were less frequently evaluated as markers of DPN were the thickness and proportion of curved stromal nerves [31], fiber reflectivity [32], fiber size (i.e., width and area) [33], and fractal dimension (which represents the measure of the structural complexity of the sub-basal nerve plexus, appearing to be reduced in DPN) [34], but it has been demonstrated that the most reliable parameter for the detection of abnormality of small fiber morphology is CNFL, which appears to be the most reproducible and strongly associated with the different measures of neuropathy severity [35,36]. We believe that nervous beading frequency, mirroring the metabolic activity of the corneal fibers, represents a great biomarker of peripheral nerve damage, probably being even more accurate than the measures of density or length. However, we are aware that nervous beading is a difficult parameter to obtain since it requires a considerable level of expertise, and it may be affected by subjectivity; thus, a method to standardize this kind of measure as well would be useful for the future. CNFL showed an even greater ability to differentiate between diabetic individuals with and without neuropathy when standardized for tortuosity [37]. In a cohort of 89 T1DM patients, the threshold value that optimized sensitivity and specificity for ruling in DPN, according to clinical and electrophysiological examinations, was a CNFL of ≤14.0 mm/mm^2^ (sensitivity 85%, specificity 84%) [38]. In a larger population composed of both T1DM (516 subjects) and T2DM (482 subjects) patients, the derivation AUC for CNFL, representing diagnostic accuracy, was 0.77 in T1DM and 0.68 in T2DM [39]. The optimal threshold for automated CNFL was 12.5 mm/mm^2^ in T1DM and 12.3 mm/mm^2^ in T2DM. In the total cohort, a lower threshold value below 8.6 mm/ mm^2^ to rule in DPN and an upper value of 15.3 mm/mm^2^ to rule out DPN were associated with 88% specificity and 88% sensitivity. 

**Figure 4:**
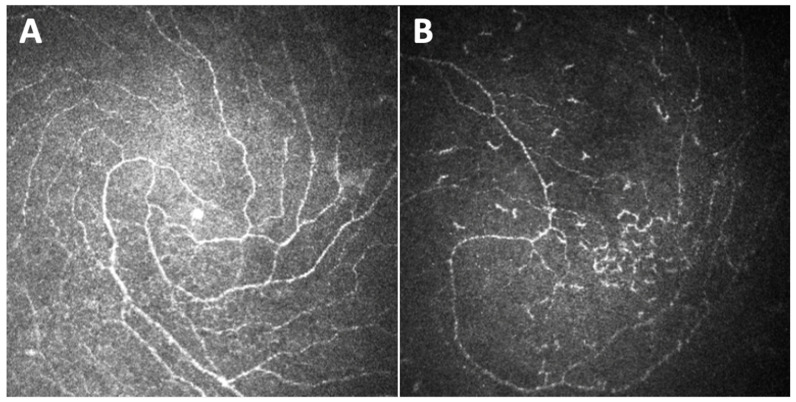
Images of the inferior whorl captured with Heidelberg Retina Tomograph III Rostock Corneal Module (Heidelberg, Germany) of a healthy subject (**A**) and a diabetic subject with diabetic peripheral neuropathy (**B**), showing an evident depletion of fibers in the diabetic neuropathic patient.

Great attention has also been given to the inferior whorl (IW), identified as an area with a vortex-like pattern located inferior and slightly nasal to the corneal apex. Given that DPN is a length-dependent, symmetrical neuropathy with the initial involvement of the most distal sensory nerves, several authors hypothesized that in such a disease, the fibers of the IW, being the most distal part of the sub-basal plexus, could be depleted before the more proximal central nerves (Figure 4). Indeed, it was demonstrated that IW length (IWL) has a comparable [40,41,42] or even greater [43] ability to diagnose patients with DPN compared with the other extensively used corneal parameters and that IWL depletion is particularly useful in the longitudinal assessment of corneal nerve loss in diabetic neuropathic patients [44]. 

An issue regarding the reliability of corneal nerve parameters as markers of neuronal damage in diabetic patients could be the influence of age on their status. However, the majority of the works searching also for a possible influence of age on the studied corneal parameters showed that the enrolled subjects’ age at the moment of examination did not correlate with the corneal sub-basal nerve plexus parameters under evaluation. This involved not only the most frequently used parameters such as corneal nerve fibers’ length and density and corneal branching density [39,45,46] but also the nerve fiber tortuosity and corneal nervous beadings [28,29,47], both in patients and in healthy control group [48], as well as in both adults and pediatric populations [49]. These findings are consistent with other reports, focusing on healthy subjects, that demonstrated how the morphology of the corneal sub-basal nerve plexus is not influenced by age, thus allowing the exclusion of a relevant biologic parameter when evaluating changes in the corneal sub-basal nerve plexus [50,51]. Conversely, few other authors evaluating corneal nerve damage in diabetic patients found a correlation between patients’ age and CNFL, CNFD, and CNBD [52,53,54,55], in accordance with papers reporting that age is a weak determinant of corneal nerve fiber abundance in healthy people [56,57].

## 4. Comparisons between CCM and other DPN Diagnostic Measures

Since the first demonstrations of corneal nerve damage in patients with DPN, CCM has been proposed as a definitive surrogate marker of neuropathy in diabetic patients, to supply the request of regulatory authorities for clinically relevant surrogate end points. These would be vital to accurately define at-risk patients, anticipate deterioration, and assess the efficacy of new therapies. Indeed, the detection of early signs of neuropathy may allow intervention with treatments to slow or reverse this condition. Although diagnostic methods such as electrophysiology, quantitative sensory testing (QST), and the assessment of neurological disability are advocated to define neuropathic severity, they have relevant limitations due to, respectively, their inability to detect small fiber dysfunction, their limited availability, and high subjectivity [58,59]. An objective evaluation of small nerve fiber damage, which is an earlier indicator of peripheral neuropathy whereas large fiber deterioration develops later, can be conducted by means of sural nerve or skin biopsy, nevertheless, these techniques are invasive and non-repeatable [60,61]. On the contrary, CCM is able not only to detect small fiber changes but also to do so non-invasively, in a very precise and reproducible way.

Several studies have compared the ability of CCM and skin biopsy to quantify small nerve fiber pathological changes to diagnose and assess the progression of DPN, demonstrating comparable diagnostic efficiency between intraepidermal nerve fiber (IENF) parameters and corneal nerve parameters, with the latter providing the significant further advantage of revealing damage before detectable nerve dysfunction in an entirely non-invasive way [62,63,64]. The diagnostic efficacy of CCM has also been tested in comparison to corneal esthesiometry, with evidence that not only do they have comparable diagnostic utility for DPN but also that CCM is definitively better and more reproducible to detect nerve damage earlier and quantitatively better [48,65,66].

The LANDMark study was specifically designed with the purpose to investigate the utility of corneal nerve morphology and function as markers of diabetic neuropathy [36]. The authors evaluated 231 individuals with diabetes with predominantly mild or no neuropathy and 61 controls by means of CCM, comparing corneal nerve fiber parameters with established tests of DPN, in particular diabetic neuropathy symptom score, neuropathy disability score, testing with 10 g monofilament, QST (warm, cold, vibration detection) and nerve conduction studies. They found that CNFL shows the strongest associations with other diagnostic tests of neuropathy as well as with established risk factors for neuropathy. Furthermore, in a sub-set of 38 T1DM individuals who fulfilled a strict criterion of “normal” classification for all seven measures of neuropathy at the baseline and were followed over a 4-year period, corneal nerve morphology, as captured by CCM, demonstrated the greatest and most sustained degeneration, among all the other measures of DPN [67].

## 5. Role of CCM in the Longitudinal Assessment of DPN

One of the greatest advantages that a diagnostic technique should have is the capacity to also detect variations in time in order to monitor the disease under evaluation. As regards CCM and corneal nerve parameters as DPN markers, several longitudinal studies have investigated changes in sub-basal nerve plexus morphology and its relationship with the conventional measures of neuropathy in individuals with diabetes. Dehgani et al. followed a T1DM patient cohort over a period of 4 years [52], and more recently, Dhage et al. enrolled both T1DM and T2DM patients for a mean follow-up of 6.5 years [68]. The first study, based on a reported association between corneal parameters and DPN severity, hypothesized that individuals with diabetes and DPN would demonstrate a faster deterioration of sub-basal nerve plexus tissue than those without DPN. The authors found that, in the DPN group, the parameter that underwent the most marked reduction over time was CNFD and suggested that branch damage might represent the primary pathological change in DPN, whereas CNFD (a parameter related to the major nerve trunks) deterioration occurs later. The reduction in CNFD, along with a non-significant decline in the other two parameters, may suggest the degeneration of major nerve trunks with concomitant regeneration reflected by an increase in the CNBD and CNFL. The study of Dhage et al. also identified over a long follow-up period a worsening of diabetic neuropathy by means of CCM, demonstrating a reduction in CNFD, but also CNBD and CNFL [68].

Longitudinal studies are extremely important since they allow the identification of the threshold values of a measure that could be used to predict the development of the disease for which the measure is employed. This also occurred with CCM and sub-basal nerve plexus parameters. Since CNFL has been demonstrated to be the most reliable parameter for the detection of abnormality of small fiber morphology, this parameter was compared between diabetic patients who did or did not develop DPN after a long follow-up period, and the receiver operator characteristic (ROC) curve was used to determine its capability to predict DPN, both in T1DM populations [69,70,71] and in larger T1DM and T2DM cohorts [72]. It was demonstrated that CCM could predict the 4-year incident DPN with 63% to 82% sensitivity and 69% to 74% specificity for the CNFL threshold cutoff of 14.1 mm/mm^2^ to 14.9 mm/mm^2^, according to this previous study.

Lewis et al. identified a new marker of DPN onset and progression, the rapid corneal nerve fiber loss (RCNFL) [73]. The authors aimed first to determine the reference distribution for the annual change in CNFL in healthy control patients, and then, from this distribution, to find a threshold for abnormal loss, and finally to apply this reference threshold to diabetic individuals, determining the prevalence of abnormal loss in a large cohort of patients. The RCNFL was defined by values exceeding the 5th percentile of 6% loss, and it was found to occur in 17% of diabetic patients. The RCNFL may thus identify patients at the highest risk for the development and progression of DPN.

## 6. How Glycemic Parameters Affect Corneal Nerve Fibers

It is well-known that, in T1DM patients, long-standing hyperglycemia is an important causative factor of neuropathy, and that vascular risk factors, such as body mass index (BMI), dyslipidemia, and hypertension are involved in the development of neuropathy independently from hyperglycemia [74,75]. However, even with good glycemic and risk factor control, the consequences of DPN can be severe. Furthermore, the results regarding a possible neurological improvement in DPN are inconclusive, probably because of the lack of appropriately sensitive and effective methods to evaluate the effect of glycemic control on DPN. Through CCM, several studies have evaluated how changes in glycemic parameters could affect corneal nerve fibers. 

A group of studies demonstrated, in a population of T1DM patients that underwent simultaneous pancreas and kidney transplantation, thus reaching euglycemia, an increase in CNFD and CNFL at 6 months, an increase also in CNBD at 12 months, and the maintenance of this improvement at 36 months [76,77,78]. Such improvement in corneal nerve fibers shown by CCM was not accompanied by significant improvement in other neuropathy assessing measures including neurophysiology, QST, corneal sensitivity, and intraepidermal nerve fiber density. Another study based on an interventional strategy that confirmed the regeneration of corneal nerve fibers after good glycemic control was that of Azmi et al., who concluded that continuous subcutaneous insulin infusion, which is superior to multiple daily insulin injections for reducing HbA1c, was also superior in promoting small-fiber regeneration, as assessed by CCM [79]. Thus, CCM results are able to track the recovery of DPN after interventions such as simultaneous pancreas and kidney transplantation and continuous subcutaneous insulin infusion.

Additionally, many observational studies conducted both on T1DM patients and on streptozotocin-induced diabetic C57Bl/6J mice reported a correlation between the glycemic control as expressed by levels of HbA1c and corneal nerve parameters, in particular, CNFD and CNFL but also CNBD and beading frequencies [36,53,80,81,82,83,84].

In recent years, the concept of glycemic variability has emerged as a risk factor for both the macrovascular and microvascular complications of diabetes, including neuropathy [85]. Diabetic patients with similar mean glucose or HbA1c levels often exhibit different microvascular and neuropathy outcomes, and glucose variability, representing the number and degree of glucose excursion, could be responsible for these complications. Mahelkova et al., starting from this assumption, tested for possible associations between parameters of glycemic compensation and corneal sub-basal nerve fiber status [45]. They found that corneal parameters were not correlated with HbA1c but instead with glycemic variability expressed as glycemic standard deviation (SD) and that they were surprisingly higher in those with higher glycemic variability. Furthermore, CNFD CNFL and CNBD resulted in significantly higher values in those with a higher total dose of insulin per kilogram, concurrent with the findings indicating that, in the peripheral nervous system, insulin facilitates nerve regeneration and that insulin applied to the cornea of diabetic mice prevent axonal loss in the sub-basal plexus [86]. Thus, the higher total dose in patients with higher glycemic variability may explain the better status of the corneal nerve fibers [45]. Another study assessing glycemic variability indexes found that the all-time SD of HbA1c was independently associated with CNFD, CNFL, and fractal dimension [87].

As regards T2DM, various works investigated the correlation between HbA1c and the corneal nerve morphology via CCM, concluding that the regeneration of corneal nerve fibers follows an improvement in glycemic control, with a significant association over time between HbA1c levels and corneal parameters, including bead size and corneal nerve thickness [30,88,89,90].

## 7. CCM Findings Help Defining Diabetic Corneal Neuropathy Pathogenesis

CCM has been revealed to be a useful tool also for the study of the pathogenesis of corneal neuropathy in diabetic subjects since the underlying basis for corneal nerve damage is not definitely established.

It has already been stated that the development of diabetic neuropathy is associated with poor glycemic control and with the development of microvascular complications, and a number of metabolic risk factors such as lipids, blood pressure, and BMI have also been shown to be related to the development of DPN. There is also evidence that an immune-mediated mechanism may act in concert with hyperglycemia to damage sensory and autonomic neurons [91]. Tavakoli et al. tested the involvement of this immune-mediated mechanism in corneal diabetic neuropathy by evaluating the presence and density of corneal dendritic cells (DCs) in a diabetic population and searching for a correlation between DCs and the extent of corneal nerve damage [92]. DC density was significantly increased in diabetic patients, particularly in those with no or mild neuropathy, whereas it decreased in those with moderate and severe neuropathy, though still remaining above control values. The authors hypothesized that DCs, at least in the early phase of nerve damage, could play a role independently of hyperglycemia since no correlation was found between HbA1c and DC density. The study of Leppin et al. had the same purpose, and to achieve it, the authors measured DC density and corneal parameters in mice models of T1DM and T2DM [93]. They found a significant negative correlation between DC density and nerve fibers in diabetic mice, concluding that the increase in DCs could play an initial role in the manifestation of diabetic corneal neuropathy. The increase in DCs upon diabetic induction might be interpreted as a cellular response to inflammation, as diabetes is known to be associated with systemic inflammation. The close position of DCs and single nerve fibers of the sub-basal nerve plexus may suggest communication between the two cell structures. As diabetes is an inflammatory disease, the interaction of the two cell types could lead to neurogenic inflammation, resulting in the release of neuropeptides, thus contributing to a bidirectional interaction of corneal DCs and nerve fibers. In conclusion, these data provide support for a potential immune-mediated basis of corneal nerve damage.

It has also been hypothesized that the pathogenesis of corneal nerve damage could have something in common with that of the retinal neurodegeneration seen in diabetes. There is mounting evidence that neurodegeneration has an important role in the pathophysiology of diabetic retinal disease and may be present even in the absence of vascular pathology [94]. There are few studies comparing alterations in corneal nerves with retinal neuropathy-linked parameter modifications in the early stages of DM2 [90,95]. They found a reduction in both corneal nerve parameters and in retinal nerve fiber layer (RNFL) and inner macular layer thickness of diabetic patients, but no variable of the corneal nerve fiber morphology was statistically significantly correlated with the thickness of any of the retinal layers in the macular or peripapillary region. It has been hypothesized that small corneal nerve fibers and retinal ganglion cells have different morbidity due to their different types of nerve structures. Taken together, the results of these studies indicate that nerve fiber degenerations in the cornea and retina occur as two independent pathological changes in diabetes, thus excluding the possibility of common pathogenesis, maintaining the assumption that nerve fiber alterations in the sub-basal nerve plexus of diabetic corneas represent the first evidence of sub-clinical DPN and appear to progress in parallel with the disease.

Furthermore, diabetic corneal nerve fiber changes have been suggested to be related to the progression of diabetic retinopathy (DR). Several studies have demonstrated, mainly but not only in T2DM populations, a strong association in the progress of DR with diabetic corneal neuropathy, which, in turn, seems to run in parallel with DPN, showing that CCM variables progressively decreased with the increasing severity of DR [31,32,48,96,97,98,99]. Some of these works also disserted the possibility that treatment with retinal argon laser photocoagulation in diabetic patients with proliferative DR could affect in a certain way the corneal sub-basal plexus determining a more pronounced decrease in CCM parameters, but the results were contrasting [32,98,100]. Bitirgen et al., beyond demonstrating a correlation between the severity of DR and that of corneal damage, also observed a significant reduction in CNFD, CNBD, and CNFL in patients without DR [98]. This, therefore, demonstrates that diabetic neuropathy in the form of corneal nerve fiber damage occurs before the development of diabetic retinopathy, in agreement with the previous data [101,102,103].

It can be argued that the parallel progress of entities such as DR and diabetic corneal neuropathy is based on their common pathogenetic mechanisms. Hyperglycemia observed in diabetic patients seems to represent the leading factor for the pathogenesis of macrovascular and microvascular complications, including neuropathy. Chronic hyperglycemia seems to be the leading factor for the pathogenesis of macrovascular and microvascular complications of diabetes, including neuropathy, leading to a metabolic cascade that causes peripheral nerve injury through an increased flux of the polyol pathway, accumulation of advanced glycosylation end products, excessive release of cytokines, activation of protein kinase C, and exaggerated oxidative stress [104,105,106].

We have to keep in mind the avascular nature of the cornea; hence, CCM parameters may not be dependent on vascular changes. The corneal nerves are nourished mainly by the aqueous humor via diffusion and partly by the axonal flow, which comes from the nerve cell body located in the brainstem; therefore, corneal nerves are not likely to have direct ischemic damage caused by macrovascular impairment, especially arterial stiffness and structural sclerosis [107]. Given this assumption, the aforementioned effects of glycemic control on corneal parameters denote the predominant role of the metabolic mechanisms for the pathogenesis of corneal nerve damage and may also reflect the impairment of metabolic support for axons normally provided by Schwann cells or the deleterious effect of diabetes on unmyelinated nerves [53]. Indeed, in histological specimens of corneas of rats with streptozocin-induced diabetes, Ishida et al. observed irregular patches of thickening and thinning in the basal lamina of Schwann cells, as well as occasional axonal degeneration in unmyelinated corneal fibers and irregular distribution of nerve beading [108]. The authors hypothesized that, in diabetes, the metabolic support for the axon normally provided by Schwann cells could be impaired, thus providing the basis for the development of corneal neuropathy. In particular, two studies demonstrated, both in T1DM and T2DM, a correlation between corneal morphological changes and peripheral nerve fibers’ function represented by axonal excitability measurements [109,110]. Since axonal excitability reflects the biophysical properties of axons, these results highlight the structural and metabolic changes in Schwann cells in diabetes, supporting the concept that axonal degeneration could be partly due to the impairment of Schwann cells.

## 8. CCM in Pediatric Population Studies

In recent years great interest has grown in the assessment of corneal nerve damage in the diabetic pediatric population. Sub-clinical DPN has been reported in 25% of newly diagnosed and 50% of children within 5 years from the diagnosis of T1DM; however, the disease is often asymptomatic in children, and early diagnosis is difficult [111]. For this reason, a technique able to easily and non-invasively detect the early signs of DPN, as CCM has been demonstrated to accomplish in adults, is fundamental even in diabetic children and young adults. In vivo CCM image analysis demonstrated good reproducibility with excellent intra- and interindividual variability in pediatric subjects, giving further evidence of the robustness of CCM as a rapid and non-invasive approach for the detection of early neuropathy in children with diabetes [112].

A study by Szalai et al. on T1DM young patients versus healthy controls reported significantly lower CNFD, CNBD, CNFL, and corneal total branch density (CTBD) and greater nerve fiber width, with these changes being more severe in patients with DR [113]. From the same population, a group of subjects that underwent a 2-year follow-up visit was subsequently enrolled to search for the progression of corneal nerve fiber abnormalities [114]. The authors found a significant decrease in CNBD and CTBD, representing the distal branches, in patients without DR and a reduction in CNFD, representing the more proximal nerves, in patients with DR, relating this result to the retrograde process of neurodegeneration typical of the DPN. The same conclusion was drawn by Ferdousi et al. showing, in a large cohort of children with T1DM, a significant reduction in CNBD and CNFL with preserved CNFD and tortuosity [115]. The reduction in CNBD and CNFL may represent the earliest pathology to the most distal nerves, sparing the more proximal major nerves represented by CNFD. The retrograde process of neurodegeneration in corneal neuropathy had already been demonstrated by a greater reduction in IWL, compared with central CNFL [43]. Gad et al. used this parameter in the assessment of corneal sub-basal nerve plexus damage in children with T1DM, demonstrating an IWL reduction beyond a reduction in other corneal parameters including CNFD, CNBD, and CNFL [49,116]. Gotze et al. reported, in otherwise healthy pediatric T1DM patients compared with controls, a reduction only in CNFL with no significant alterations in other parameters [117]. 

Therefore, these few studies conducted in young T1DM patients have produced inconsistent results, probably because of the limited sample size, with low statistical power for detecting differences between patients and healthy controls. A large cohort of T1DM children and adolescents has been more recently studied, demonstrating a consistent reduction in fiber length and density parameters, providing substantial evidence that early alterations in small corneal nerve fibers are detectable in children and adolescents with diabetes [118]. These findings highlight the possible need for the earlier screening of diabetic neuropathy in children with T1DM using CCM.

## 9. Limitations and Future Perspectives

Throughout this review, we highlighted how CCM has been demonstrated to be a useful tool for the detection of sub-basal nerve plexus damage in diabetic patients in a reliable and non-invasive way. Furthermore, it has been demonstrated that this type of nerve damage correlates with the severity of DPN and that CCM has the ability to detect it earlier and with the same or even greater diagnostic accuracy than other known neuropathy measures, which instead present limitations such as subjectivity and invasiveness. Unfortunately, the use of CCM remains under-utilized and too limited to research purposes rather than application in standard clinical practice.

Just recently, CCM has been applied in two randomized clinical trials: One confirmed CCM sensitive enough to detect the superior efficacy of 8-week mecobalamin intramuscular injection treatment for DPN compared with the oral tablet treatment [119]. The other, the BOND study, which is still ongoing, aims to assess the effects of treatment with benfotiamine, compared with placebo, in participants with T2DM and mild-to-moderate symptomatic DPN, with the primary endpoint being a change in CNFL after 12 months of treatment and secondary endpoints including other CCM measures [120].

However, one of the major problems limiting the extension of CCM to standard diabetes clinical practice is image analysis. A variety of methods for quantifying sub-basal plexus parameters have been used in the past several years, often differing from study to study. Originally, researchers used manual analysis of sub-basal corneal nerve parameters, but it was a tedious, subjective, and time-consuming procedure. Considerable expertise was necessary to quantify corneal nerve changes. To be clinically useful as a diagnostic tool, it is essential that CCM images are automatically analyzed and the different nerve parameters quantified.

ImageJ, particularly with its plugin NeuronJ, currently represents one of the most frequently used methods to conduct a semi-automated analysis of the sub-basal nerve plexus, since it has been used in multiple studies to assess parameters such as IWL, CNFL, CNFD, CNBD, beading frequencies, and corneal nerve thickness [30,42,48,84,88,90,93,119]. It is a validated open-source image analysis platform developed by the National Institutes of Health (NIH; Bethesda, MD, USA), which includes NeuronJ, a semi-automated nerve-tracing plugin that intuitively draws a line over the center of a visible nerve fiber as the nerve is traced [121,122,123].

**Figure 5:**
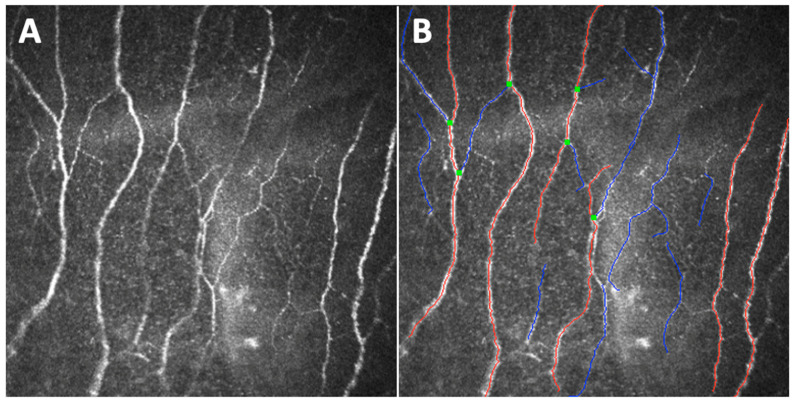
An example of elaboration via ACCMetrics software (**B**) of an image of the sub-basal nerve plexus captured with Heidelberg Retina Tomograph III Rostock Corneal Module (Heidelberg, Germany) from the central cornea of a healthy subject (**A**). The elaborated image shows main fibers in red, branches in blue, and branching points in green.

Ruggeri et al. made the first step toward the development of an automated method of analysis (using Confoscan 4) that does not require any user intervention, designing a model for the automatic recognition and tracing of corneal nerves in confocal microscopic images [124,125]. Efron et al. tested a semiautomated nerve analysis software on images captured with Heidelberg Retina Tomograph 3 with Rostock Corneal Module to measure CNFL [126]. All measures presented highly repeatable results. Then, Malik et al. began to develop an image analysis software that allows an automatic quantification of some selected, unfortunately not all, corneal nerve parameters (i.e., the previously cited CNFD, CNBD, CNFL, and CTBD, as well as corneal nerve fiber area and corneal nerve fiber width) from single or multiple CCM images: the ACCMetrics software (Figure 5) [127,128,129]. Other studies confirmed and validated the reliability of corneal nerve fiber measurements via ACCMetrics; in particular, the automated quantification of CNFL, CNFD, and CNBD has demonstrated comparable DPN detection ability to manual and semi-automated analysis [63,130,131]. Midena et al. have recently demonstrated, integrating the ACCMetrics with nerve beading quantification, the presence of corneal neuropathy in subjects previously affected by the COVID-19 disease [17].

Nevertheless, the standard criteria for CCM image analysis are still missing. Future perspectives include methods based on artificial intelligence. A number of fully automated deep learning methods based on convolutional neural networks (CNNs) have recently been developed to analyze corneal nerve parameters, in both animals [132,133] and humans. Models such as U-net [133,134,135,136], CNS-Net [137], and deepNerve [132,138] have been used as multi-step approaches to segment and trace corneal nerves from CCM images and, therefore, process their properties. Deep learning algorithms have also been applied to build automatic corneal nerve tortuosity grading systems with the purpose of replacing the time-intensive and perceptually biased subjective tortuosity grading [136,139]. Recently, a few studies proposed deep learning models based on CNNs to directly associate CCM images to healthy subjects or to those with diabetic neuropathy, demonstrating great accuracy. Moreover, several authors demonstrated the superior performance of CNNs, compared with ACCMetrics, in state-of-the-art paradigms, thus revealing its potential in identifying clinically useful features [134,135,140].

## 10. Conclusions

In a world in which the incidence of diabetes is continuously increasing, and the macrovascular and microvascular complications of this disease are incurring an enormous financial burden, early diagnosis aimed to successfully prevent or monitor invalidating complications is essential. DPN represents one of the most frequent complications of diabetes, affecting approximately 50% of diabetic patients. Several methods used to identify and follow this condition, including electrophysiology, QST, assessment of neurological disability, and sural nerve or skin biopsy, have different and significant limitations since some of them are unable to detect small fiber dysfunction and are highly subjective, while others are invasive and non-repeatable. CCM, providing a non-invasive quantification of corneal nervous small fibers, which represent part of the peripheral nervous system, has been demonstrated to be a valid in vivo tool to detect early sub-basal nerve plexus damages in adult and pediatric diabetic patients, correlating with the severity of DPN. Reviewing the current literature, we investigated the utility of CCM in assessing DPN through an exploration of different aspects of the technique. We focused on the different corneal parameters used to evaluate sub-basal nerve plexus, among which CNFL is probably the most reproducible and strongly associated with other measures of neuropathy severity, and on the corneal nerve changes that directly follow glycemic or other metabolic parameters’ improvement as well as deterioration. We also evaluated the use of CCM as an instrument to better understand the pathogenesis of corneal diabetic neuropathy, which seems to be correlated with an immune-mediated mechanism but independent from retinal neurodegeneration, which also occurs in the first phases of diabetes. Unfortunately, despite its great potential, CCM has still limited application in daily clinical practice, mainly because of the lack of knowledge among doctors taking care of diabetic patients. Probably, the introduction of artificial intelligence in the evaluation of CCM data will break this limiting barrier.

## Data Availability

No new data were created or analyzed in this study. Data sharing is not applicable to this article.
